# Supplementary material for: Substantial Na-Ion Storage at High Current Rates: Redox-Pseudocapacitance through Sodium Oxide Formation
Source: Nanomaterials (Basel). 2022 Nov 30;12(23):4264. doi: 10.3390/nano12234264 (PMC9737611; doi:10.3390/nano12234264)
Supplement: Supplementary file 1 [file nanomaterials-12-04264-s001.zip › nanomaterials-2021082-supplementary.pdf]

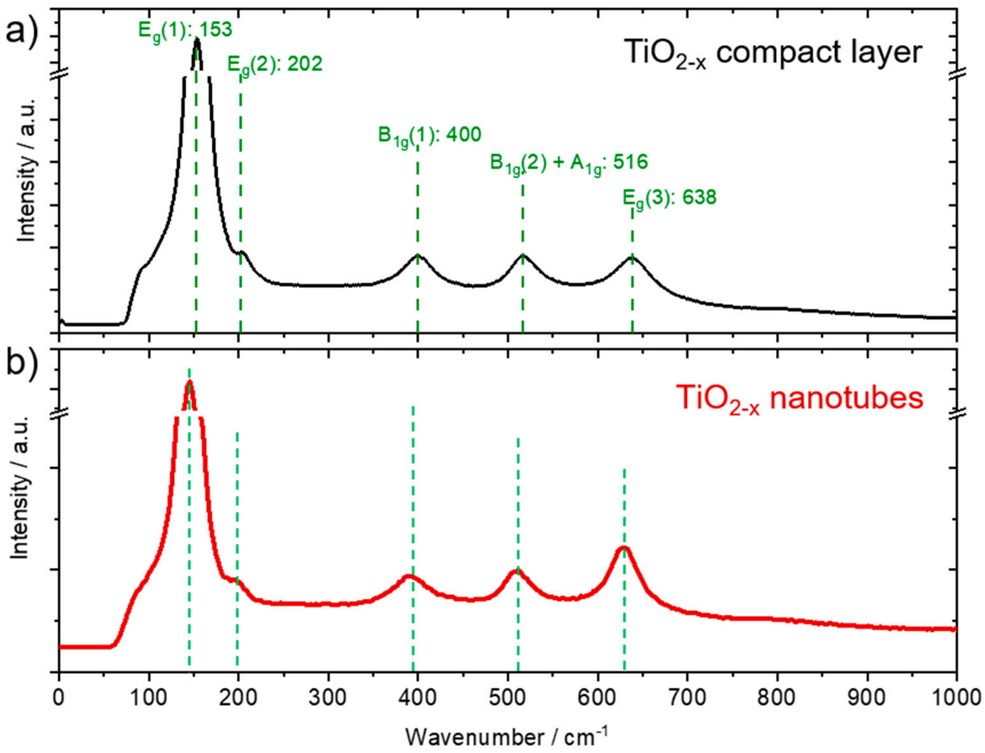

Figure S1. Measured Raman spectra of (**a**) compact TiO_{2-x} layer and (**b**) self-organized TiO_{2-x} NTs.

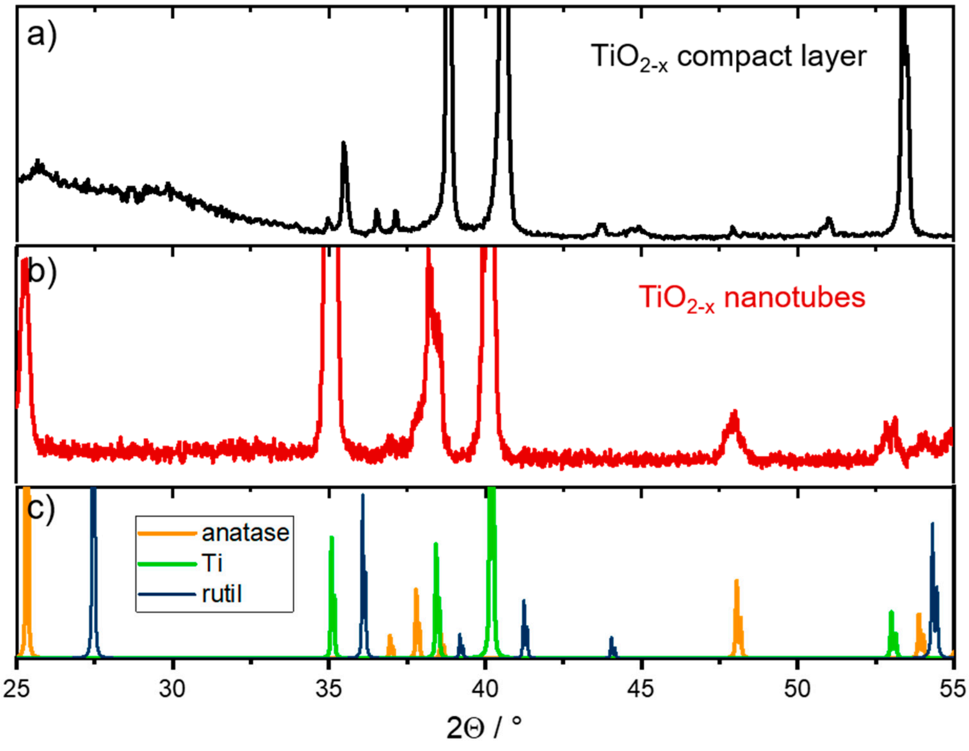

Figure S2. Measured XRD patterns of (**a**) compact TiO_{2-x} layer and (**b**) self-organized TiO_{2-x} NTs. (**c**) Reference data for Ti metal, anatase and rutil.

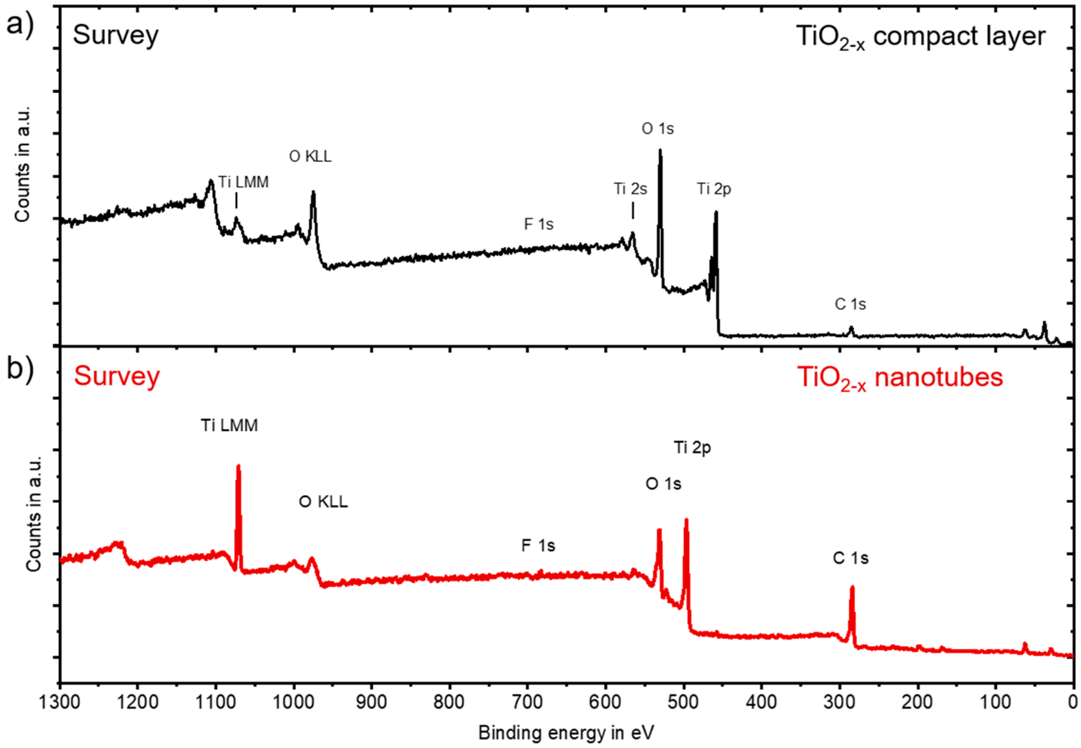

Figure S3. Measured XPS survey spectra of (**a**) compact TiO_{2-x} layer and (**b**) self-organized TiO_{2-x} NTs.

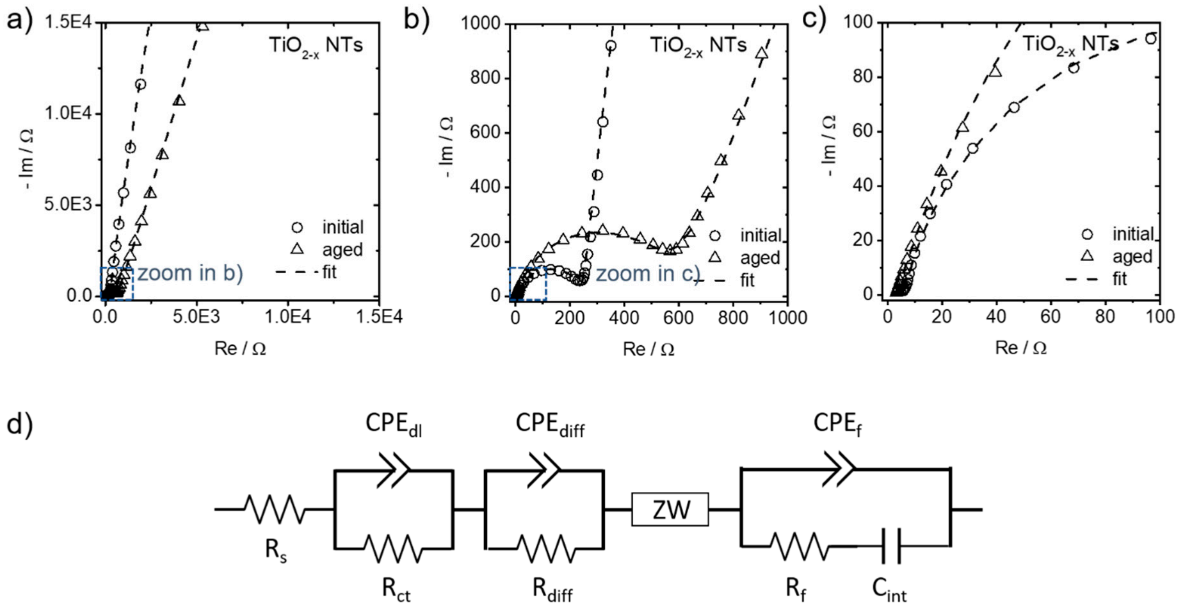

Figure S4. Electrochemical impedance spectroscopy (EIS). Nyquist plots of anatase TiO_{2-x} NTs before (initial) and after (aged) film formation by 230 galvanostatic sodiation/desodiation cycles, plotted from 100 kHz to 7.4 mHz in three different magnifications (**a–c**). (**d**) Equivalent electric circuit used for fitting the EIS data. R_s: solution resistance, $R_{ct\,1}$ interfacial Ti/TiO_2 NTs electron charge transfer resistance, $C_{dl\,1}$ and C_{dl}: corresponding capacitances, ZW: Warburg element for semi-infinite diffusion, (capacitances modelled with a CPE).

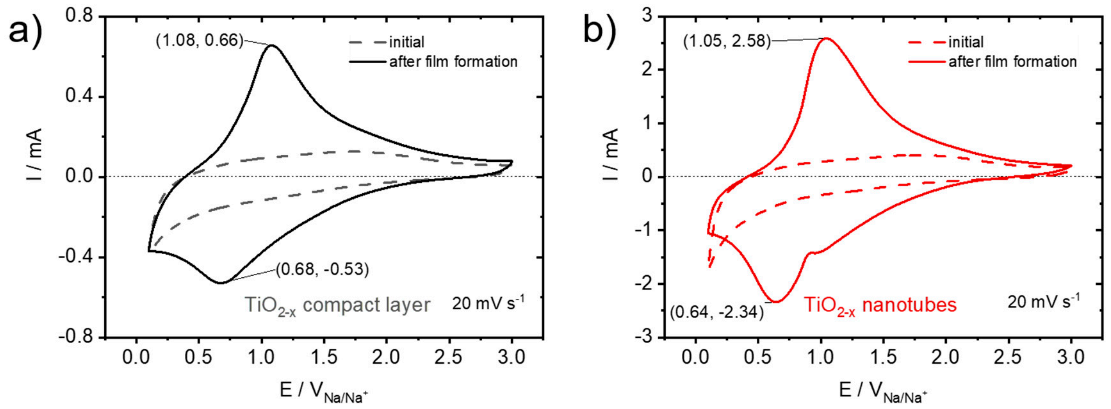

Figure S5. Electrochemical characterization and comparison. CV measurements for the initial (dashed line) and aged (solid line, after film formation) electrodes with (**a**) a compact TiO_{2-x} layer and (**b**) self-organized TiO_{2-x} NTs at a scan rate of 20 mV s^{-1}.
